# Artificial-goosebump-driven microactuation

**DOI:** 10.1038/s41563-024-01810-6

**Published:** 2024-02-09

**Authors:** Mingchao Zhang, Aniket Pal, Xianglong Lyu, Yingdan Wu, Metin Sitti

**Affiliations:** 1 Physical Intelligence Department, Max Planck Institute for Intelligent Systems, Stuttgart, Germany; 2 Institute of Applied Mechanics, University of Stuttgart, Stuttgart, Germany; 3 Institute for Biomedical Engineering, ETH Zürich, Zürich, Switzerland; 4 State Key Laboratory of Robotics and System, Harbin Institute of Technology, Harbin, China; 5 School of Medicine and College of Engineering, Koç University, Istanbul, Turkey

**Keywords:** Liquid crystals, Applied physics

## Abstract

Microactuators provide controllable driving forces for precise positioning, manipulation and operation at the microscale. Development of microactuators using active materials is often hampered by their fabrication complexity and limited motion at small scales. Here we report light-fuelled artificial goosebumps to actuate passive microstructures, inspired by the natural reaction of hair bristling (piloerection) on biological skin. We use light-responsive liquid crystal elastomers as the responsive artificial skin to move three-dimensionally printed passive polymer microstructures. When exposed to a programmable femtosecond laser, the liquid crystal elastomer skin generates localized artificial goosebumps, resulting in precise actuation of the surrounding microstructures. Such microactuation can tilt micro-mirrors for the controlled manipulation of light reflection and disassemble capillary-force-induced self-assembled microstructures globally and locally. We demonstrate the potential application of the proposed microactuation system for information storage. This methodology provides precise, localized and controllable manipulation of microstructures, opening new possibilities for the development of programmable micromachines.

## Abstract (web summary)

Light-induced artificial goosebumps on liquid crystal elastomer skin are used to precisely manipulate passive microstructures, achieving a localized and controllable system for programmable micromachines.

## Main

Microactuators, with their capability to precisely control and manipulate small-scale objects and systems, enable the development of miniature microelectromechanical systems (MEMS)^1–3^ for applications in miniature robotics^4,5^, biomedical devices^6^ and integrated electronics^7^. Magnetically responsive microactuators have been widely embraced due to their facile fabrication process, rapid response times and large range of motion^1,8–10^. Other actuation strategies, such as thermal, photonic or electrical actuation, often rely on using intricate active materials like hydrogels^11,12^, liquid crystal elastomers (LCEs)^13^, conducting materials^2,3^ and piezoelectric materials^14^. These versatile materials exhibit great adaptability to various environments, expanding the applicability across different scenarios. Incorporating anisotropic properties within these smart materials is often essential to achieve specific motions or boost output actuation^15–18^. For example, spatially programmed heterogeneous magnetic profiles enable complex two-dimensional and three-dimensional (2D and 3D) motions in polymeric microactuators^18^. Similarly, the alignment of molecular structures within LCE microactuators enables complex non-reciprocal motions^17^. However, achieving precise manipulation of spatial anisotropy within these synthetic microactuators presents substantial fabrication challenges at such small scales^19^. Furthermore, the motion modes of these microactuators are usually fixed once the anisotropy is programmed after fabrication and cannot be easily tuned or reconfigured without altering the structure or properties of the microactuators^20^, limiting their versatility and adaptability. Moreover, controlling movements in individual or site-specific microactuators is necessary to achieve the complex motions seen in many natural microorganisms^21,22^, which presents a further challenge.

Developing individually controlled microactuators not only offers important benefits in understanding the complex and collective behaviour of natural microorganisms, but also provides a powerful tool for manipulating microstructures. In the realm of microstructure fabrication within MEMS, a common challenge arises from elastocapillary coalescence driven by capillary forces^23–26^, often resulting in unwanted structural distortion or even destruction^27–29^. To mitigate these issues, researchers often resort to using rigid materials for constructing small structures and optimizing them without relying on large-aspect-ratio designs^30,31^, or avoid crossing the liquid-to-gas phase boundary during development^32^. Although these preventive strategies can partially preserve structural integrity, they limit the versatility of the fabricated structures^33^ or increase fabrication challenges and costs due to the stringent requirements (such as precise control in pressure and temperature) in the case of the critical point drying process. Furthermore, while capillary-induced self-assembly has been effectively harnessed to create visually appealing ordered patterns in some cases^23,34^, its use beyond aesthetics has been restricted, primarily due to the absence of efficient tools capable of post-processing and manipulating the assembled structures at such small length scales, either globally or locally. Therefore, the pursuit of microactuation systems capable of selectively or comprehensively reversing self-assembled structure formation, without compromising design and material versatility, holds great promise for practical applications.

To tackle these challenges, we propose a light-fuelled active LCE skin driving passive 3D-printed microstructures on the skin, inspired by the actuation mechanism of fine biological hairs standing on the skin when stimulated. Such a microactuation system offers an efficient approach for local or global manipulation of microstructures. Passive microstructures are fabricated by inactive photoresists via two-photon polymerization (2PP) on a light-responsive artificial skin made of a tri-network LCE. When exposed to a femtosecond (fs) laser, the LCE film generates localized microscale artificial goosebumps, which induce a local curvature that causes the targeted passive microstructures to move. By precisely programming the laser trajectories, speed and power, we achieve two degrees of freedom (2-DOF) of motion control of the microstructures, including 0–360 degrees of rotation. Using such a bionic microactuation system, we demonstrate its capability in tilting micro-mirrors to precisely steer light reflection, and in locally disassembling capillary-induced microstructure self-assemblies. Furthermore, we show that these well-controlled assemblies can serve for information encoding, highlighting the potential of our microactuation system for information storage applications.

## Concept of a light-powered bionic microactuation system

**Fig. 1:**
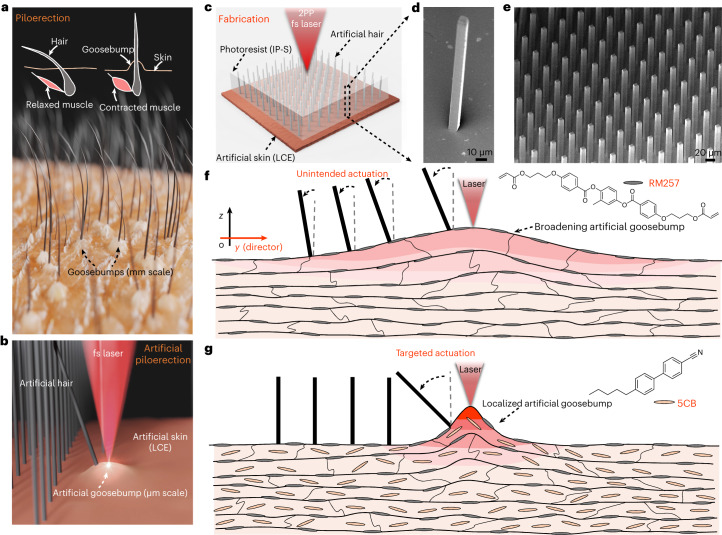
Artificial-goosebump-driven microactuation system. **a**, Schematic illustration showing the phenomenon of fine hairs standing on the skin upon stimulation and the underlying mechanism of goosebumps. **b**, Schematic illustration of the piloerection-inspired microactuation system, where the goosebumps, generated by the local laser heating, induce a temporary local curvature that deflects the 2PP-printed passive microhairs. **c**, Schematic illustration showing the fabrication process of artificial polymeric (IP-S photoresist) microhair arrays using 2PP on a tri-network LCE skin. **d**, Scanning electron microscopy (SEM) image displaying a 2PP-printed microhair (with a width of 8 µm and height of 150 µm) on the LCE skin. **e**, SEM image showing a 2PP-printed microhair array on the LCE skin. **f**,**g**, Schematic illustrations presenting the mechanism involved in artificial goosebump generation for the microactuation, where a broadening of an artificial goosebump causes unintended actuation of microstructures (**f**), while a localized goosebump brings about precise and targeted actuation (**g**). RM257, 1,4-bis-(4-(3-acryloyloxypropyloxy)benzoyloxy)-2-methylbenzene; 5CB, 4-cyano-4′-pentylbiphenyl.

Goosebumps, also known as piloerection or horripilation, are a common physiological response of the body to certain stimuli, including strong emotions, certain sensations or cold temperatures^35^. This response causes the biological hairs on the skin to stand up straight or bristle (Fig. 1a). Upon stimulation, the tiny muscles connected to the hair follicles contract, leading to the formation of goosebumps and causing the hairs to elevate. This reaction can make animals appear larger and more intimidating to potential predators, or it can create a layer of trapped air for thermal insulation, helping regulate body temperature^36^. Inspired by such goosebump-based biological hair actuation, we propose a microactuation system consisting of two components: a passive microstructure (for example, artificial hair) array driven by a deformable active elastomeric artificial skin (Fig. 1b). The microstructure arrays are 3D-printed onto the artificial skin surface via a 2PP process using a commercially available photoresist (IP-S; Fig. 1c). This process enables the fabrication of high-density microstructure arrays (Fig. 1d,e). The active/dynamic artificial skin is made of a uniaxial-aligned tri-network LCE, capable of providing local deformation to actuate the selected individual microstructures. We employ a fs laser, which not only enables the 2PP-based fabrication of these microstructures with submicrometre-scale resolution (down to 100 nm), but also allows precise and 2-DOF motion of these microstructures (Fig. 1b).

Upon exposure to the laser locally, the LCE artificial skin generates temporary microscale artificial goosebumps due to its photothermal adsorption. Local heating triggers a phase transition from nematic to isotropic alignment at the hot spot, resulting in a vertical expansion and formation of an artificial goosebump. Such a goosebump generates a local microscale curvature, deflecting the microstructures on top of the artificial skin for actuation. However, a substantial challenge lies in the precise control of this actuation, due to the difficulties in effectively focusing the generated heat at the targeted spot on the LCE skin. The inherent nematic alignment of the LCE skin ensures vertical deformation (resembling goosebumps) when stimulated, but it also gives rise to a high thermal conduction along the direction of polymer chain alignment (director). This high thermal conduction poses a hurdle in concentrating the heat exactly where needed, resulting in a broader artificial goosebump than desired and causing unintended actuation of the microstructures in a wide area (Fig. 1f). By contrast, through the design and optimization of the LCE network chemistries, we achieved the formation of sharp and localized goosebumps and realized the targeted actuation of the microstructures (Fig. 1g), which holds promise for practical applications that demand precise manipulation at small length scales.

## Fabrication of the LCE artificial skin

**Fig. 2:**
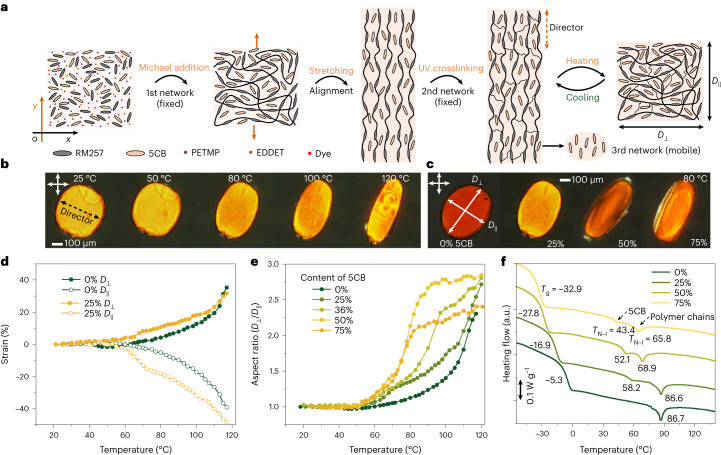
Thermal actuation of the tri-network LCE film. **a**, Schematic illustration depicting the fabrication process of the tri-network LCE. PETMP, pentaerythritol tetrakis(3-mercaptopropionate); EDDET, 2,2-(ethylenedioxy)diethanethiol. **b**, Cross-polarized optical images displaying the evolution of a typical LCE (with 25 wt% dopant, small mobile liquid crystal molecules, 5CB) at different temperatures. **c**, Cross-polarized optical images showing the LCE samples with varying amounts of 5CB at 80 °C. *D* with subscripts (∥ and ⊥) denotes the lengths of the LCE measured along (∥) and perpendicular to (⊥) the director. **d**, Anisotropic shape changes of the LCE samples with and without 25 wt% 5CB dopant. **e**, Anisotropic deformations of the LCE samples with different amounts of 5CB dopant. **f**, Differential scanning calorimetry measurements conducted on the LCE samples with different dopants. For LCE without doping, two peaks include the glass transition temperature (*T*_g_) and nematic-to-isotropic transition temperature (*T*_N–I_) of the crosslinked bi-network of LCE. For LCE samples with doping, the three peaks observed can be attributed to the *T*_g_ and *T*_N–I_ of the dopant inside the LCE, and the *T*_N–I_ of the crosslinked bi-network LCE. Source data

The fabrication process of the LCE artificial skin is adopted from the classic two-step thiol-Michael LCE chemistries^37–40^, and we modified our previously reported recipe^13^ by incorporating mobile liquid crystal molecules (4-cyano-4′-pentylbiphenyl (5CB), as a typical compatible nematogenic solvent) for a coupled tri-network to achieve a reduced actuation temperature and enhanced localization in deformation (detailed fabrication process in the Methods). The LCE artificial skin is composed of uniaxially aligned, crosslinked bi-networks with small, mobile 5CB liquid crystal molecules embedded within them (forming a tri-network, as shown in Fig. 2a), which act as a plasticizer to adjust the intrinsic properties of the LCE^41–43^. The obtained LCE film (with a thickness of ~100 µm) exhibits high anisotropic deformation upon heating. Upon heating, it undergoes reversible shrinkage along the alignment direction (director) and simultaneous expansion perpendicular to its director during the transition from a nematic (ordered) to an isotropic (disordered) alignment. To help visualize the anisotropic deformation, circular shapes are cut from the LCE film, and they can be seen to gradually transform into an elliptical shape during the heating process (Fig. 2b).

The inclusion of mobile small liquid crystal molecules (5CB) within the crosslinked bi-networks (Fig. 2c) remarkably decreases the actuation temperature of the LCE film. We recorded the deformation of the films as a function of temperature and observed that the critical temperature for the onset of deformation and the temperature at which the maximum deformation rate (*D*′) occurs decrease with increasing 5CB content (Fig. 2d). The aspect ratio of the LCE film (*D*_⊥_/*D*_∥_) with different amounts of 5CB dopants increases from 1 (initial circular shape) as the temperature increases. Generally, the temperatures corresponding to *D*′ and saturation deformation decrease with increasing 5CB content (Fig. 2e). This behaviour can be attributed to the fact that these small molecules create spacing between the crosslinked polymer chains, thereby increasing the free volume and reducing interchain and intrachain interactions, leading to lower actuation temperatures^44–46^. The plasticization effect of 5CB molecules can be evidenced by the differential scanning calorimetry results (Fig. 2f), where the glass transition temperature (*T*_g_) and the nematic-to-isotropic transition temperature (*T*_N–I_) of the LCE decrease with an increase of 5CB. Nevertheless, it’s important to emphasize that an excessive introduction of 5CB (beyond 50 wt%) not only makes the resulting LCE film more prone to yield and permanent plastic deformation (details in Supplementary Note 1 and Supplementary Fig. 1), but also diminishes its actuation performance (resulting in a lower extention of deformation). The fabricated LCE film demonstrates a rapid response to temperature fluctuations (Supplementary Video 1) and undergoes muscle-like contraction and expansion.

## Generation of localized artificial goosebumps by laser

**Fig. 3:**
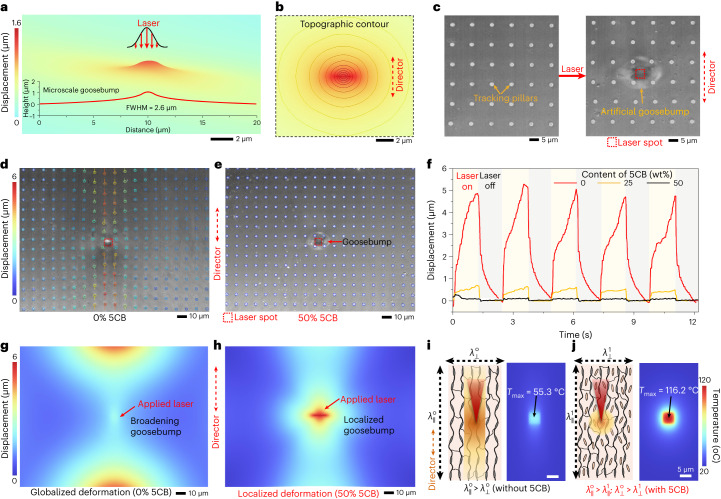
Generation of local artificial goosebumps on the LCE skin via a fs laser. **a**, FE simulation illustrating the generation of an artificial microscale goosebump using a laser with a scanning area of 1 × 1 µm^2^. **b**, Topographic contour displaying the FE-simulated microscale goosebump. **c**, Optical images showcasing the goosebump generated on the LCE surface, where arrays of 2PP-printed micropillars (diameter, 1 µm; height, 2 µm; array spacing, 10 µm) are used to track the displacement of the LCE surface (using a laser with a scanning area of 5 × 5 µm^2^). **d**, Experimental tracking of surface displacement on a LCE surface without 5CB dopant actuated by a laser with a scanning area of 5 × 5 µm^2^. **e**, Experimental tracking of surface displacement on an LCE surface with a 50% 5CB dopant. **f**, Displacement evolution of a tracking micropillar (located 50 µm along the director direction from the laser centre) on the LCE with varying amounts of 5CB. **g**, FE simulation demonstrating the displacement of the LCE surface without 5CB dopant, actuated by a laser with a scanning area of 5 × 5 µm^2^. **h**, FE simulation showing the displacement of the LCE surface with 50% 5CB dopant, actuated by the same laser. **i**,**j**, Schematics (left) and FE simulations in temperature distribution (right) of the LCE without (**i**) and with (**j**) 5CB dopant, actuated by the same laser. It indicates that the decrease in thermal conduction of the LCE, resulting from the doping of small mobile molecules, contributes to the localization of laser heating and induces large and site-specific local deformations (artificial goosebumps). *T*_max_ is the simulated maximum temperature generated in the spot. λ is the thermal conductivity with the superscripts (0 and 1) representing the LCE without (0) and with (1) 5CB dopant, respectively. The subscripts (∥ and ⊥) denote measurements taken along (∥) and perpendicular to (⊥) the director. Source data

**Extended Data Fig. 1:**
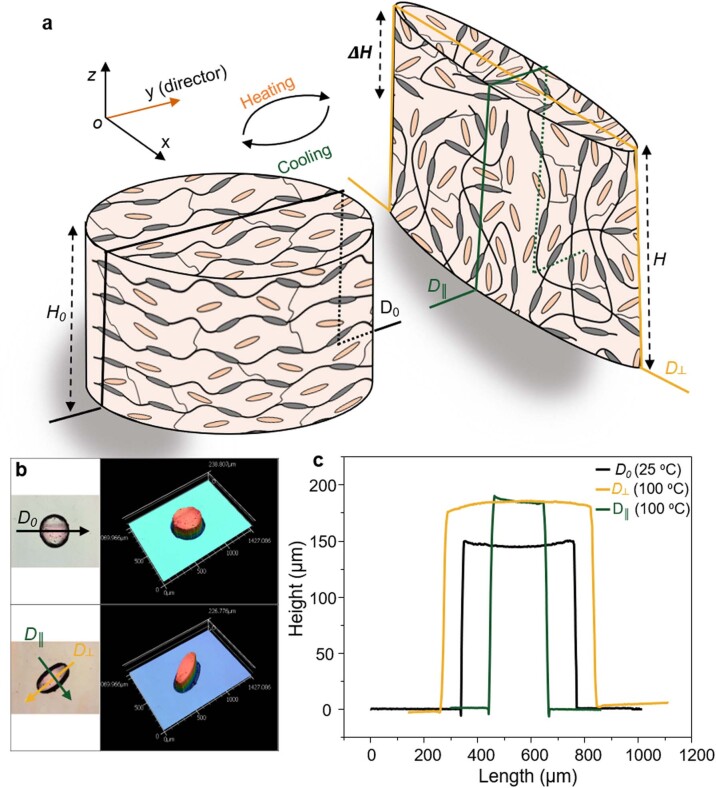
Spatial deformation (in x, y, and z direction) of the LCE. **a**, Schematic illustration showing the spatial deformation of the LCE. **b**, Optical images and constructed 3D images of the LCE before and after heating. **c**, Height profiles of the LCE along and transverse to the director at 25 ^o^C and 100 ^o^C. Source data

The LCE film, when heated globally, exhibits deformation not only in the planar direction (*x* and *y*), but also along its thickness direction (*z*) as a result of the nematic-to-isotropic transition of the uniaxial polymer chains (Extended Data Fig. 1a,b). This deformation in the *z* direction (Extended Data Fig. 1c) allows for the generation of localized vertical bumps through site-specific laser heating. Finite-element (FE) simulations are carried out to help us understand the generation of microscale artificial goosebumps on the LCE film (simulation details can be seen in the Methods). The results of the simulations, as depicted in Fig. 3a, reveal that a laser with a scanning area of 1 × 1 µm^2^ can induce a microscale bump with a full-width at half-maximum (FWHM) of 2.8 µm around the spot. The elliptical shape of the bump can be attributed to the anisotropic deformation as revealed in Fig. 3b. Specifically, the director direction undergoes shrinkage while the transverse direction experiences expansion. This elliptical shape also matches the experimentally observed bump shown in Fig. 3c.

The objective is to generate localized bumps via the focused laser spot for site-specific and precise actuation of the microstructures, while minimizing unwanted deformations to the surrounding areas (Fig. 1f,g). We discovered that incorporating 5CB molecules contributes to the sharp localization of the resulting bumps on the surface. By printing arrays of small micropillars on the LCE skin, we could track the surface motion of the LCE skin as a function of different loadings of 5CB using a laser with a scanning area of 5 × 5 µm^2^ (Fig. 3c). For the LCE sample without any 5CB doping, remarkable displacement (with an hourglass-like distribution) can be observed over a large area (Fig. 3d and Supplementary Video 2). By contrast, the displacement field falls off sharply outside the laser scanning area for the doped LCE samples of 25 wt% 5CB (Supplementary Fig. 2) and 50 wt% 5CB (Fig. 3e). Figure 3f illustrates the displacement of a typical micropillar located 50 µm along the director direction from the laser centre, showing that increasing the doping amount of 5CB notably localizes the surface displacement induced by the laser.

The sharp localization of the induced bumps on the laser spot can be attributed to the reduced thermal conductivity (*λ*) resulting from the doping of 5CB molecules. We performed FE simulations of the laser-induced displacement on the LCE films with varying amounts of 5CB doping. The displacement mapping from the simulation results closely matches the experimental results depicted in Fig. 3d,e. Without 5CB doping, the laser induces a wide distribution of displacement around the spot (Fig. 3g), while with doping, a sharp focus of displacement is observed surrounding the induced bumps (Fig. 3h). The nematic alignment of polymer chains provides continuous channels for efficient phonon propagation, contributing to high heat conduction along the chain direction, while resistance (phonon scattering) is higher in the perpendicular direction, causing a remarkable thermal conductivity anisotropy^47^. However, the doped mobile nematogenic molecules within the networks readily undergo reorientation or rearrangement upon heating, impeding heat diffusion (Fig. 3i). Consequently, thermal conduction along both directions is notably diminished. The simulated temperature mappings depicted in Fig. 3i demonstrate that the generated temperature at the laser area is lower in the LCE film without doping than in the doped film (Fig. 3j). This discrepancy arises from the ability of the mobile molecules to inhibit heat diffusion, thereby contributing to sharp deformation (that is, a localized artificial goosebump) at the laser.

## Passive microactuation by the artificial goosebumps

**Fig. 4:**
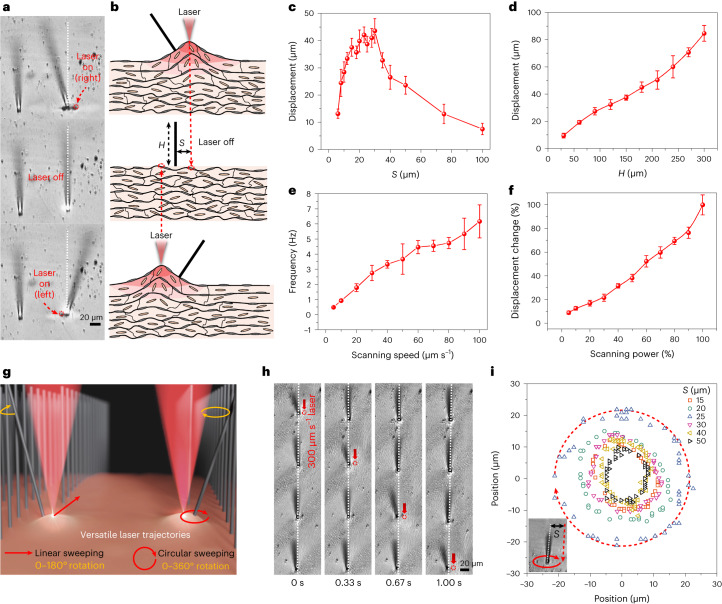
Light-powered goosebump-driven passive microhairs with 2-DOF motion. **a**, Optical images depicting the deflection of microhairs driven by goosebumps powered by a fs laser at a scanning area of 5 × 5 µm^2^. **b**, Schematic illustration demonstrating the actuation of microhairs through goosebump-induced deformations. **c**, Displacement of the microhair tip when actuated by a laser at different spacings (*S*) between the laser centre and the microhair. Data points are shown as mean ± s.d. (*n* = 15). **d**, Displacement of the microhair tip with varying heights (*H*) when actuated by the same laser. Data points are shown as mean ± s.d. (*n* = 15). **e**, Frequency response of the hair motion driven by different laser scanning speeds. Data points are shown as mean ± s.d. (*n* = 15). **f**, Displacement extent of the microhair tip actuated by a laser with different scanning power levels. Data points are shown as mean ± s.d. (*n* = 15). **g**, Schematic illustration showcasing the free trajectories of the laser, enabling 2-DOF motions of the microhair tip. **h**, Snapshots of optical images illustrating a moving laser along a row of microhairs, sequentially inducing their motion. **i**, Trajectories (0–360° rotation) of the microhairs actuated by circular sweepings of the laser surrounding the microhairs with different spacings, with one example shown in the inset. Source data

**Extended Data Fig. 2:**
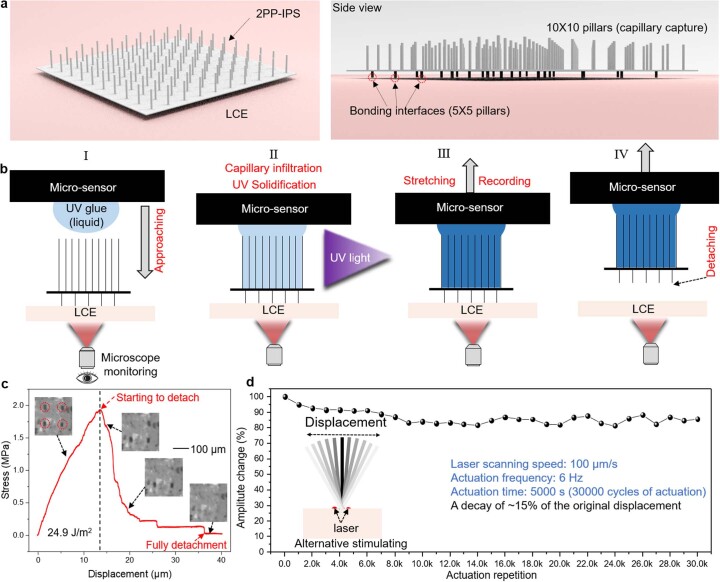
Quantitative analysis of interfacial bonding strength and long-term actuation of microstructures. **a**, Schematic of a printed architecture for detachment test. The architecture (IP-S) is composed of two vertical arrays of micropillars separated by a plane. The bottom micropillars (5×5 array; height: 50 µm; spacing: 400 µm) was printed to the LCE substrate, and the top dense and long micropillars (10×10 array; height: 200 µm; spacing: 150 µm) are designed to capture UV-curable glue (P/N6801, Norland Inc.) on the probe (connected to the load cell) through capillary infiltration. The plane is designed to prevent the contact of UV-curable glue with the bottom micropillars (as a separation barrier). **b**, Schematics of the detachment test process: (I) UV-curable glue was applied to the probe (connected to the load cell), and the probe slowly approached the sample at a speed of 5 μm/s and stopped as soon as the UV glue wetted and infiltrated into the top dense micropillars; (II) a 365 nm UV lamp (UV-15 S/L, Herolab) was employed to cure the glue for a duration of 20 min; (III) the probe began to ascend at a rate of 100 nm/s; (IV) the stretching leaded to sequential detachment of these micropillars, and finally they were completely detached from the LCE substrate. **c**, Profile of stress vs. stretching displacement during detachment. The insets are real-time captured optical images showing the micropillars are sequentially detached from the LCE substrate. **d**, Actuation displacement change of a micropillar subjected to the actuation of 30,000 cycles under a high frequency of 6 Hz. The inset is the schematic showing the actuation of a micropillar with a high frequency of actuation. Source data

A firm interfacial bonding between the 3D-printed microstructures and the LCE surface ensures their robust actuation (Supplementary Fig. 3, Extended Data Fig. 2 and the analysis of the interfacial bonding in the Methods contain further details). The generation of microscale artificial goosebumps induces local curvature on the LCE surface, causing deflection in the surrounding microhairs that are 3D-printed on the LCE surface. The deflection of the microhairs always occurs away from the laser (Fig. 4a and Supplementary Video 3). Once the laser is withdrawn, the microhairs return to their original standing state. By applying the laser alternately around the two sides of the microhairs, we can observe the sequential generation of bumps on both their sides, resulting in their swinging motion, as shown in Fig. 4b. In addition, through subjecting a microhair to over 30,000 cycles of actuation at a high frequency of 6 Hz, we observed a modest decay of approximately 15% in the actuation amplitude (Extended Data Fig. 2d). This slight decay can be attributed to the ageing effects of the LCE polymers during the actuation process.

Since the microhairs are 3D-printed on the LCE surface and remain aligned with the normal vector of the surface, the geometry of the generated microbumps notably affects their actuation performance. Several parameters of the microactuation system, including the spacing (*S*) between the laser centre and the microhairs, the height (*H*) of the printed microhairs, the scanning speed of the laser and the scanning power of the laser, are investigated. The displacement of the tip (using a laser with a scanning area of 5 × 5 µm^2^) increases with increasing *S*, reaching a peak at *S* = 25 µm, and then decreases as *S* is further increased (Fig. 4c). This trend can be attributed to the fact that the slope is zero at the centre and far edges of a bump, while the maximum slope is located between these two points. Moreover, for the same deflection angle, the tip displacement of the microhairs increases linearly with the increase in *H* as shown in Fig. 4d. Additionally, the actuation frequency and extent of the microhairs can be controlled by adjusting the scanning speed and power of the laser. Higher scanning speeds result in higher actuation frequencies of the microhairs (Fig. 4e), while a higher scanning power leads to a larger deflection amplitude (Fig. 4f).

Due to the high programmability (laser details in Supplementary Note 2) of the laser trajectory, speed and power, we could realize a 2-DOF motion of the microhairs. For example, when the laser sweeps linearly beside a microhair, the tip undergoes a rotation from 0 to 180 degrees with varying amplitudes. Circular sweeping of the laser around a microhair causes it to rotate with angles ranging from 0 to 360 degrees, as depicted in Fig. 4g. Specifically, the laser’s free trajectories can sweep through a microhair array in any direction (at a speed of 300 µm s^–1^), enabling sequential activation of semicircular rotations in these microhairs (Fig. 4h, Supplementary Fig. 4 and Supplementary Video 4). Furthermore, circular sweeping of the laser with different distances (*S*) centred at the microhairs induces their circular rotation with varying amplitudes (Fig. 4i and Supplementary Video 5), following the same trends as shown in Fig. 4c.

## Applications of the passive microactuation systems

**Fig. 5:**
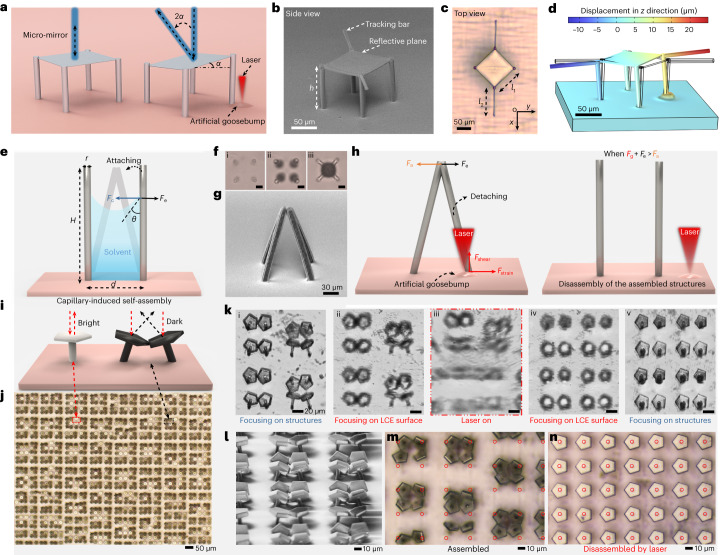
Applications of the artificial-goosebump-driven microactuation systems. **a**, Schematics illustrating micro-mirrors with for controllable light steering. **b**,**c**, SEM (**b**) and reflective optical (**c**) images of a 3D-printed micro-mirror with dimensions *l*_1_ = *l*_2_ = *h* = 100 µm. **d**, FE simulation illustrating the plane tilt of the micro-mirror driven by the generated artificial goosebump on the LCE skin. **e**, Schematic illustrating the process of capillary-induced self-assembly of printed high-aspect-ratio (slender) microstructures. *r* is the diameter of the slenders, *d* is the spacing distance of surrounding slenders and *θ* is the contacting angle of liquid and slenders. When the elastic restoring force (*F*_e_) cannot resist the adhesion (*F*_a_) of the slenders, the assembly is thus formed. **f**, Optical images of an example self-assembly during the liquid solvent evaporation process (development process). Scale bars are 30 µm. **g**, SEM image displaying the self-assembled microstructure. **h**, Schematic illustration demonstrating the disassembling mechanism of the assembled structure using the laser-driven goosebumps, which generate a disturbance force (*F*_g_), such as shear (*F*_shear_) or strain (*F*_strain_), that overcomes the cohesion (*F*_a_) of the self-assembled structures, resulting in their disassembly. **i**, Application of the disassembling mechanism by employing a mushroom-like mirror as the basic assembling unit. When the micro-mirrors are assembled, light is scattered, leading to a dark view (black dashed box as in **j**), while disassembled mirrors reflect light, resulting in a bright view (red dashed box as in **j**). **j**, Optical images showing the 3D-printed mushroom-like mirror arrays. **k**, Optical images capturing the disassembling process of the assembled structures using a laser. Scale bars are 20 µm. **l**,**m**, SEM image (**l**) and reflective optical image (**m**) showing the assembled microstructures. **n**, Reflective optical image displaying the disassembled structure after the laser treatment. The red circles indicate the bottom positions of the mushroom-like mirror before (**m**) and after (**n**) the laser treatment.

**Extended Data Fig. 3:**
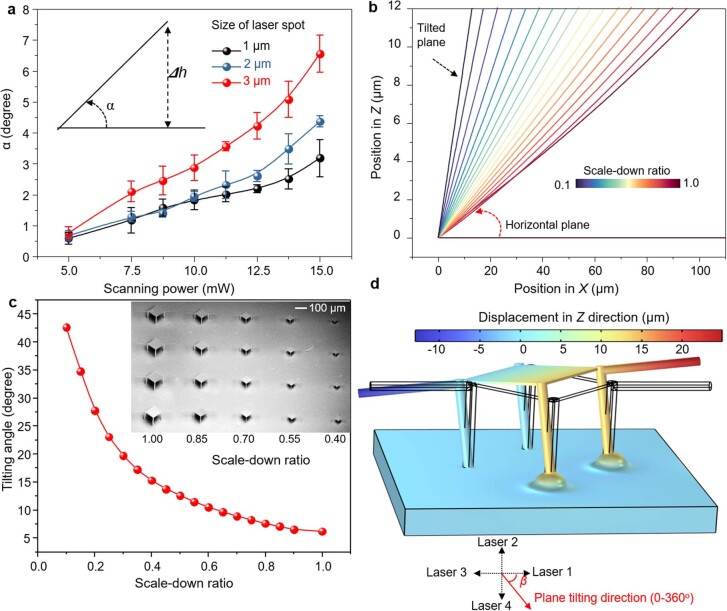
Experimental and simulation results of artificial-goosebump-driven micro-mirrors for controllable light steering. **a**, Experimental results demonstrating the tilting angle (α) of the mirror plane as a function of scanning power and the size of the laser spot. Data points are shown as mean ± s.d. (*n* = 6). **b**, Simulated plane positions of the micro-mirrors with different scale-down ratios driven by the same artificial goosebump, where the lateral size of the plane is set to be 100 µm for the micro-mirror with a scale-down ratio of 1. **c**, Simulated titling angles as a function of the scale-down ratios of the micro-mirrors. The inset is the SEM image of the printed micro-mirrors with different scale-down ratios. **d**, Simulation depicting the tilting direction of a micro-mirror subjected to two simultaneously applied laser spots.The bottom schematic shows any tilting direction of a micro-mirror can be achieved by cooperatively actuating the four supporting legs. Source data

Our microactuation systems, driven by artificial goosebumps, offer versatile capabilities for various practical applications by combining global and local actuation of microstructures. One notable application is the fabrication of micro-mirrors designed for precise control of light reflection (Fig. 5a). These micro-mirrors consist of a reflective plane supported by four supporting pillars or legs, with motion-tracking bars on each side, directly printed onto the LCE skin (Fig. 5b,c). The introduction of a laser-induced artificial goosebump results in a lifting or upward motion in the micro-mirror’s pillar, leading to a controlled tilting (degree and direction) of the reflective plane and redirection of incident light (Fig. 5d, Extended Data Fig. 3, Supplementary Fig. 5 and Supplementary Video 6, and details in Supplementary Note 3).

Our microactuation systems, as another example, can be employed to disassemble capillary-force-induced self-assembled microstructures globally and locally. The elastocapillary interaction between solvent and structures often leads to undesirable distortion or even structural failure (Fig. 5e). As the evaporation progresses, the influence of capillary force (*F*_c_) becomes predominant, compelling these structures to deform and eventually adhere to neighbouring structures (Fig. 5f and Supplementary Video 7). If the elastic restoring force (*F*_e_) due to the deformation of structures cannot resist the adhesion/cohesion (*F*_a_), the self-assembled structures are thus maintained (Fig. 5g). Once these structures have attached, disassembling them efficiently becomes a formidable task due to the inherent challenges associated with manipulating small structures. Our microactuation system offers a solution for the targeted disassembly of capillary-force-induced self-assembled microstructures. By using a laser to generate artificial goosebumps on the LCE artificial skin, we can introduce disturbance forces (*F*_g_), such as shear (*F*_shear_) or strain (*F*_strain_) to the assembled structures. When the combined effect of *F*_g_ and *F*_e_ exceeds the maximum *F*_a_, the assembled structures can be released, allowing them to revert to their original design (Fig. 5h).

To show the efficacy of our approach, we employ mushroom-like micro-mirrors as the basic assembly units. These mirrors exhibit a bright appearance when they are free-standing and reflect incident light backward. However, when they are self-assembled to their surrounding counterparts through attaching at the micro-mirror edges, their tilted surfaces redirect the incident light, causing them to appear dark (Fig. 5i). Large arrays of these mushroom structures were printed on the LCE film, resulting in a random assembly pattern with a mixture of assembled structures (dark) and free structures (bright; Fig. 5j). Our microactuation method can open assemblies of various forms, including bi-, tri- and tetra-assemblies or other assemblies (Fig. 5k and Supplementary Video 8). By focusing the laser on the LCE surface (Fig. 5k(iii)), the scanning of the generated goosebumps enables the release of these assembled structures over a large area. Following the laser treatment, the assembled structures transition from a dark state (Fig. 5l,m) to a bright one (Fig. 5n), and the mirror centres realign with the bottom pillars (marked as red circles), restoring the original configuration.

**Fig. 6:**
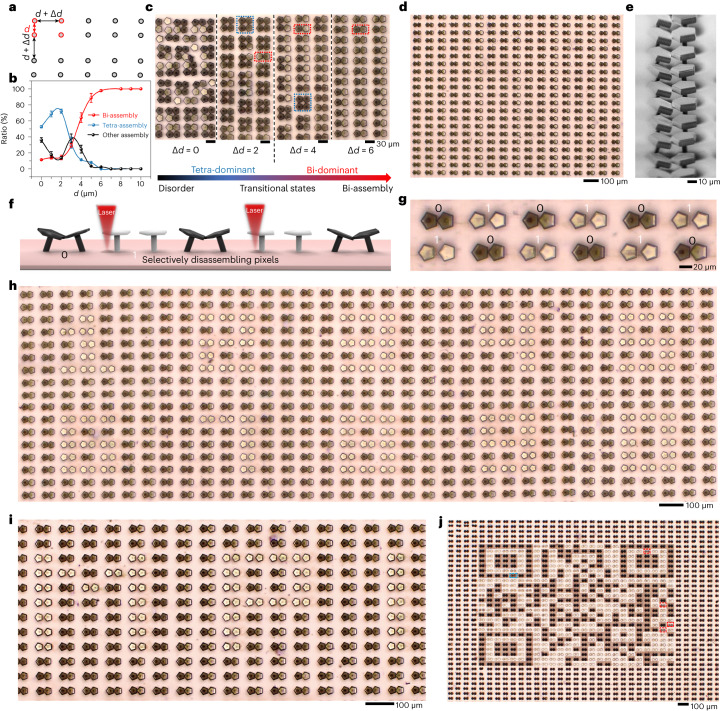
Local disassembly of uniformly self-assembled mirror-pixels for information storage. **a**, Strategy for generating uniform bi-mirror self-assemblies by adjusting the additional spacing (Δ*d*) between mirror pairs. **b**, Quantitative analysis of the yields of bi-assemblies, tetra-assemblies and other assemblies as a function of Δ*d* after being self-assembled. Data points are shown as mean ± s.d. (*n* = 12). **c**, Optical images depicting the self-assembled mirror arrays resulting from different values of Δ*d*. The blue and red dashed boxes denote tetra-assemblies and bi-assemblies, respectively. The colour bar at the bottom explains the transition of different assemblies as the increase of Δ*d*. **d**, Optical image showcasing large-area uniform bi-assemblies after being assembled. **e**, SEM image of uniform bi-assemblies. **f**, Schematic illustration showing the local disassembly of the uniform bi-assemblies, with 0 and 1 as in **g**. **g**, Optical image showing the local disassembly of the uniform bi-assemblies, where 0 stands for the assembled stage and 1 stands for the opened stage. **h**, Optical image showing written digital numbers from 0 to 9. **i**, Optical image showing written letters ‘MPI’. **j**, Optical image showing a written QR code with the content ‘Hello MPI-IS’. Defective pixels including unintended disassembly (0.16%) and unopened pixels (0.64%) are marked in red and blue rectangles, respectively. Source data

We also demonstrate the application of our microactuation method as a novel means of data storage using controllable switches between dark and bright mushroom microstructures. Prior to selectively writing information, it is crucial to achieve large areas of uniformly assembled structures. To accomplish this, we employ the bi-assembly of two mushroom structures as the basic pixel unit. Random and non-uniform assemblies can occur when mushroom structures are printed with uniform spacing and subsequently evaporated (Supplementary Fig. 6). However, by introducing an additional spacing (Δ*d*) between two paired mushroom structures and their surrounding pairs, we can achieve uniform bi-assemblies (Fig. 6a and Supplementary Video 9). By increasing the spacing, the random assemblies transform into tetra-dominant assemblies before being quickly replaced by bi-assemblies as Δ*d* increases. Ultimately, a 100% ratio of bi-assemblies is achieved when the spacing exceeds 6 µm (Fig. 6b,c). This approach enables the generation of large quantities of uniform bi-assemblies without any defects over a considerable area on the LCE surface (Fig. 6d,e).

**Extended Data Fig. 4:**
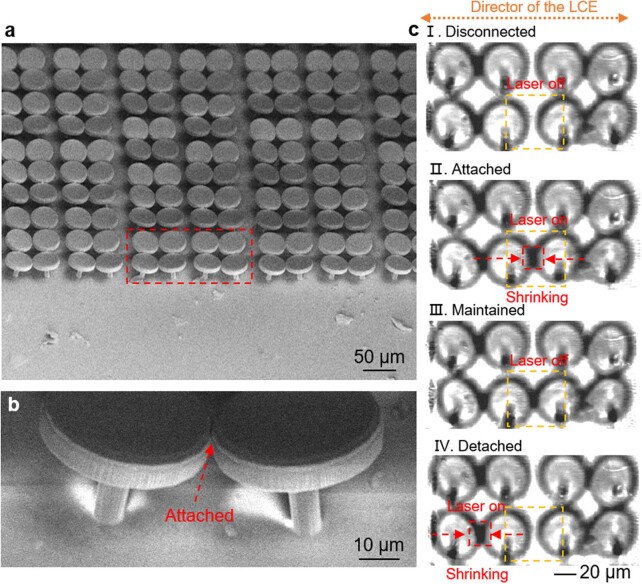
Controllable manipulation of mushroom-like microstructures as a switch by laser. **a**, SEM image illustrating an array of mushroom-like round mirrors. Round-shaped mirrors were employed to enable symmetric geometries for equal possibilities of interaction to their surroundings. **b**, SEM image showing two attached mushroom mirrors. **c**, Optical images capturing the manipulation process of controllably attaching and detaching two mushroom mirrors. By exposing the laser to the centre of two separated micromirrors along the director of the LCE (I and II), the shrinkage of the LCE along the director brings the mirrors closer, resulting in their attachment. Even after retracting the laser, the attached pair remains intact due to the cohesion between them (III). When the laser is applied to the surroundings of the attached mirrors, the resulting shrinkage at the laser induces a pulling force that separates and detaches adjacent mirror pairs (IV). This controllable on/off switching between attachment and detachment of the microstructures shows a promising micromachine application.

Within the uniform ‘canvas’ of bi-assemblies as pixels, we show that they can be both globally (Supplementary Fig. 7 and Supplementary Video 10) and locally (Fig. 6f) opened using the programmable laser. In this encoding scheme, the bi-assembly is represented as 0 (dark pixel) and its disassembly as 1 (bright pixel; Supplementary Fig. 8). Due to the sharp localization of artificial goosebumps on the LCE skin, we can open individual pixels of interest without disturbing the surrounding pixels (Fig. 6g and Supplementary Video 11). By programming various laser parameters, we can encode information into these bi-assembly pixels. Examples include writing digital numbers from 0 to 9 (Fig. 6h), alphabet letters such as ‘MPI’ (for Max Planck Institute; Fig. 6i) and patterns like alternating dark and bright rectangles (Supplementary Fig. 9). Complex patterns, such as a QR code with 625 (25 × 25) pixels containing the message ‘Hello MPI-IS’, can be written by programming the laser (Fig. 6j). There are still a few defective pixels, including unwanted disassembly (0.16%, marked in blue) and unopened pixels (0.64%, marked in red). However, with a high success rate of 99.2%, our strategy demonstrates very high effectiveness and practicality. Furthermore, the density of pixels on the LCE surface can be increased by rationally scaling down the size of each pixel (Supplementary Fig. 10), to further augment the information writing capacity. In addition, our microactuation method also leverages the unique anisotropic deformation properties of the LCE film to achieve controllable attachment and detachment of microstructures to their surroundings (Extended Data Fig. 4). These demonstrations highlight the potential of our bionic microactuation method for local manipulation of microscopic structures, and further applications in micromachines, microrobots, biomedicine, microfluidics and integrated electronics.

## Methods

### Materials

All chemicals were used without treatment, as received. The LCE precursor included RM257 (monomer, >98.0%, TCI), PETMP (crosslinker, >95.0%, Sigma-Aldrich), EDDET (flexible chain extender, >95.0%, Sigma-Aldrich), dipropylamine (thermal catalyst, 99.0%, Sigma-Aldrich), 2-hydroxy-4′-(2-hydroxyethoxy)-2-methylpropiophenone (Irgacure-2959, photocatalyst, 98.0%, Sigma-Aldrich), 5CB (nematic liquid crystal dopant, 98%, Sigma-Aldrich), 2,6-di-*tert*-butyl-4-methylphenol (thermal inhibitor, ≥99.0%, Sigma-Aldrich), 2-(*N*-ethyl-4-((4-nitrophenyl)diazenyl)anilino)ethyl prop-2-enoate (Disperse Red 1 acylate, photothermal dye, 95.0%, Sigma-Aldrich) and chloroform (solvent, ≥99.0%, Sigma-Aldrich). IP-S (Nanoscribe) was used as the inactive photoresist for fabricating the passive microstructures, and 2-propanol (≥99.0%, Sigma-Aldrich) was used as the solvent for the development.

### Synthesis of the LCE film

The fabrication process for the LCE film was modified from our previously reported recipe^13^ (Fig. 2a). Clean glass plates (5 × 5 cm^2^) were assembled with two plate spacers (200 µm) fixed at the two edges. To prepare the LCE precursor, we mixed RM257 (1,000 mg), PETMP (94.2 mg), EDDET (211.4 mg), 2 wt% dipropylamine (in chloroform, 156.8 mg), Irgacure-2959 (7.5 mg), 2,6-di-*tert*-butyl-4-methylphenol (2.7 mg), Disperse Red 1 acrylate (4.0 mg) and 5CB (in a different weight ratio to RM257; unless otherwise stated, we adopted 25 wt% of dopant) in chloroform solvent (0.4 ml). The mixture was then heated to 90 °C and dispersed using vortex mixing. The hot solution was injected into the glass chamber and kept at room temperature to allow the first crosslinking reaction of Michael addition to occur (in a dark environment). After 6 hours, a swollen polydomain gel was obtained by opening the glass chamber. It was then transferred to a vacuum oven and dried for another 6 hours (in a dark environment). The dried film was subjected to a strain of 150% of its original length for uniaxial molecular alignment. Subsequently, it was exposed to 365 nm ultraviolet light to initiate the second crosslinking process, which fixed the uniaxial alignment. During these procedures, the LCE film was collected for further use.

### 2PP-based 3D printing of microstructures on the LCE skin

The 2PP-based 3D printing of the passive microstructures on the LCE film was carried out in a cleanroom environment. The LCE film was carefully tailored into a 1 × 1 cm^2^ square and attached to a glass substrate to prevent air trapping. Prior to fabricating the microstructures, the LCE surface was cleaned by subjecting it to ultrasound inside a 2-propanol solution. Afterward, a commercial photoresist (IP-S) was applied onto the LCE surface and subjected to the 2PP process using a fs laser (Nanoscribe) for various 3D architectures. To ensure the stability of the fabricated microstructures, their aspect ratios were kept below 30. Following the development process in a 2-propanol solution, the microstructures were successfully fabricated onto the LCE surface. A layer of 20 nm gold was sputtered on the LCE surface to enable a surface plasmonic effect to enhance the photothermal efficiency of the laser. In addition, the thin layer of gold also enhances the reflection of the mushroom-like mirrors, enabling the bright view of the disassembled mirrors.

Other lithography methods, such as UV-mask lithography, are adept at creating simple passive microstructures, including artificial hairs. In terms of processing efficiency for mass production of 2D or 2.5-dimensional patterns^48^, UV-mask lithography generally surpasses the 2PP process. However, 2PP distinguishes itself in the creation of complex 3D microstructures (like mushroom micro-mirrors). It simplifies the otherwise labour-intensive and intricate procedures that are standard in UV-mask lithography for such intricate 3D designs.

### Interfacial bonding between the microstructures and LCE skin

A robust interfacial bonding between the 3D-printed microstructures and the LCE skin is essential for enabling laser actuation and facilitating subsequent applications. To ensure a strong connection, the following steps were taken (Supplementary Fig. 3a). The first step involved an incubation process where IP-S photoresist was applied to the surface of the LCE skin. Following this, an incubation time of 30 min allowed the IP-S monomer molecules to slightly swell the contacting surface. The gentle swelling occurred due to the inherent compatibility of the photoresist (IP-S, methacrylate-based monomers) and the LCE (polyacrylate-based polymers), where the IP-S monomers could slightly penetrate the LCE interface^49–51^. The laser printing process then started beneath the surface, keeping a consistent distance of 5 µm below the automatically found interface (key code, var $interfacePos = 5). Consequently, a penetrating polymer network was formed at the interface, resembling the structure of tree roots, to ensure a sturdy and durable connection (Supplementary Fig. 3b).

To measure the interfacial bonding strength, we employed a customized experimental set-up for assessing the force for detaching the printed microstructures from the LCE substrate^52^. The set-up comprises a high-resolution load cell (GSO-25, Transducer Techniques) with a resolution of 0.01 mN, mounted on a vertical, high-precision piezo motion stage (LPS-65 2″, Physik Instrumente). A camera (Grasshopper3, Point Grey Research) connected to an inverted microscope (Axio Observer A1, Zeiss) was positioned beneath the sample for real-time image recording. A strong connection of the load cell to the printed microstructure is the basis for the measurement. Therefore, we typically designed a microstructure (micropillar arrays, IP-S; Extended Data Fig. 2a) and recorded the force following a measurement process (Extended Data Fig. 2b). The stress at the interface is calculated by counting the total contact areas of the bottom micropillars with the LCE substrate.

When the stress reached 1.93 MPa, we observed the sequential detachment of these micropillars from the LCE substrate (as shown in Extended Data Fig. 2c). Complete detachment of these micropillars from the substrate allowed us to calculate an average interface bonding energy of 24.9 J m^–2^, a value notably higher (2–3 orders of magnitude) than typical van der Waals bonding^53^. Additionally, we actuated the microhairs under a high frequency of 6 Hz to assess the resilience of the interface (Extended Data Fig. 2d). Impressively, even after subjecting the microhairs to more than 30,000 cycles of actuation, they remained firmly attached to the interface, with 85% of the initial actuation amplitude still being retained. This demonstrates the formation of a robust interface that effectively resists detachment over extended periods of use.

### Characterization

Optical images were recorded via an optical microscope (Axio Imager 2, Zeiss) with reflective and cross-polarized transmissive modes. The 3D deformation of the LCE films was measured by a 3D laser scanning microscope (VK-X250, Keyence). The morphologies of the 3D-printed microhairs and mushroom-like micro-mirrors were measured using a scanning electron microscope (Leo Gemini 1530). The actuation of the microstructures was also conducted inside the Nanoscribe 2PP system by programming its fs laser (centre wavelength, 780 nm; pulse duration, 80 fs; repetition rate, 80 MHz). The real-time motions of the microstructures were recorded using a built-in AxioVision Live-View camera in the Nanoscribe system (transmissive mode), and the motions were later analysed in ImageJ software (Fiji). The phase transition temperatures of the LCE samples were determined through differential scanning calorimetry (DSC2500, TA Instruments). The mechanical properties of the LCE films were measured with a universal testing machine at a strain rate of 0.5% s^–1^ (5942, Instron).

### Finite-element simulations

FE simulations were conducted using the Heat-Transfer-Solids and Solid Mechanics modules in the software COMSOL Multiphysics 6.0. A laser with a Gaussian distribution was applied onto the LCE surface (200 × 200 × 50 µm^3^) with a certain laser scanning area (1 × 1 or 5 × 5 µm^2^). The anisotropic properties of LCE include the mechanical properties (*E*, Young’s modulus), thermal conductivity (*λ*) and coefficient of thermal expansion (*α*) along the director (molecular alignment) and its transverse directions. The LCE experiences different thermal deformations in the *x, y* and *z* directions (Fig. 2d and Extended Data Fig. 1). Shrinkage (*α*_*x*_ < 0) was observed along the director while expansion (*α*_*y*_ > 0; *α*_*z*_ > 0) was observed along the other two directions. Time-dependent simulations were conducted to obtain the laser-induced deformation and temperature distribution on the surface. As a result, both the simulated and experimentally observed artificial goosebumps were elliptical. The mechanical properties of 3D-printed architectures (such as the micro-mirror, made from IP-S) were derived from the existing literature^13^.

## Online content

Any methods, additional references, Nature Portfolio reporting summaries, source data, extended data, supplementary information, acknowledgements, peer review information; details of author contributions and competing interests; and statements of data and code availability are available at 10.1038/s41563-024-01810-6.

### Supplementary information

Supplementary Information Supplementary Notes 1–3 and Figs. 1–10.

Supplementary Video 1 Muscle-like LCE film showing fast deformation of contraction and expansion due to air-blowing-induced temperature fluctuation (heated at 100 °C, in real time).

Supplementary Video 2 Comparison of laser-induced in-plane deformation of the LCE with 5CB dopant of 0 and 50 wt% (in real time).

Supplementary Video 3 Light-fuelled passive microhairs on the LCE (in real time).

Supplementary Video 4 Sequential actuation of microhairs powered by linear sweeping of laser (in real time).

Supplementary Video 5 A 0 to 360° rotation of microhairs powered by circular sweeping of laser (in real time).

Supplementary Video 6 Artificial-goosebump-driven tilting of a micro-mirror on the LCE skin (in real time).

Supplementary Video 7 Self-assembly of printed large-aspect-ratio micropillars due to evaporation-induced capillary force (in real time).

Supplementary Video 8 Laser-enabled global disassembling process of self-assembled microstructures including bi-assemblies, tetra-assemblies and other assemblies (in real time).

Supplementary Video 9 Comparison of self-assembled mushroom-like mirror arrays with uniform spacing design (*d*) and non-uniform spacing design (in real time).

Supplementary Video 10 Laser-enabled global disassembly process of uniform bi-assembly arrays (shown five times faster).

Supplementary Video 11 Programmable disassembly process for selectively opening bi-assembly pixels for information storage (in real time).

Supplementary Data Statistical source data of Supplementary Information.

### Source data

Source Data Fig. 2 Statistical source data.

Source Data Fig. 3 Statistical source data.

Source Data Fig. 4 Statistical source data.

Source Data Fig. 6 Statistical source data.

Source Data Extended Data Fig. 1 Statistical source data.

Source Data Extended Data Fig. 2 Statistical source data.

Source Data Extended Data Fig. 3 Statistical source data.

## Data Availability

All data are available in the main text or the Supplementary Information. Source data are provided with this paper.

## References

[1] Zhang S (2023). 3D-printed micrometer-scale wireless magnetic cilia with metachronal programmability. Sci. Adv..

[2] Ren Z (2022). Soft-robotic ciliated epidermis for reconfigurable coordinated fluid manipulation. Sci. Adv..

[3] Wang W (2022). Cilia metasurfaces for electronically programmable microfluidic manipulation. Nature.

[4] Hu X (2021). Magnetic soft micromachines made of linked microactuator networks. Sci. Adv..

[5] Liu Q (2021). Micrometer-sized electrically programmable shape-memory actuators for low-power microrobotics. Sci. Robot..

[6] Ghazali FAM (2020). MEMS actuators for biomedical applications: a review. J. Micromech. Microeng..

[7] Jang K-I (2017). Self-assembled three dimensional network designs for soft electronics. Nat. Commun..

[8] Wang Z (2020). Hybrid magnetic micropillar arrays for programmable actuation. Adv. Mater..

[9] Kang M (2022). Self‐assembled artificial nanocilia actuators. Adv. Mater..

[10] Gu H (2020). Magnetic cilia carpets with programmable metachronal waves. Nat. Commun..

[11] Gao N (2021). Chemical-mediated translocation in protocell-based microactuators. Nat. Chem..

[12] Lee YW (2023). Multifunctional 3D‐printed pollen grain‐inspired hydrogel microrobots for on‐demand anchoring and cargo delivery. Adv. Mater..

[13] Zhang M (2021). Liquid‐crystal‐elastomer‐actuated reconfigurable microscale Kirigami metastructures. Adv. Mater..

[14] Li J, Liu Y, Deng J, Zhang S, Chen W (2022). Development of a linear piezoelectric microactuator inspired by the hollowing art. IEEE Trans. Ind. Electron..

[15] Gregg A, De Volder MF, Baumberg JJ (2022). Light‐actuated anisotropic microactuators from CNT/hydrogel nanocomposites. Adv. Opt. Mater..

[16] Li Z, Yang F, Yin Y (2020). Smart materials by nanoscale magnetic assembly. Adv. Funct. Mater..

[17] Li S (2022). Self-regulated non-reciprocal motions in single-material microstructures. Nature.

[18] Kim J (2011). Programming magnetic anisotropy in polymeric microactuators. Nat. Mater..

[19] Duan Y (2019). Robust thermoelastic microactuator based on an organic molecular crystal. Nat. Commun..

[20] Hagiwara Y (2023). Photothermally induced natural vibration for versatile and high-speed actuation of crystals. Nat. Commun..

[21] Shapiro OH (2014). Vortical ciliary flows actively enhance mass transport in reef corals. Proc. Natl Acad. Sci. USA.

[22] Gilpin W, Prakash VN, Prakash M (2017). Vortex arrays and ciliary tangles underlie the feeding–swimming trade-off in starfish larvae. Nat. Phys..

[23] Pokroy B, Kang SH, Mahadevan L, Aizenberg J (2009). Self-organization of a mesoscale bristle into ordered, hierarchical helical assemblies. Science.

[24] Hu Y (2020). Chiral assemblies of laser‐printed micropillars directed by asymmetrical capillary force. Adv. Mater..

[25] Liu X (2021). Capillary‐force‐driven self‐assembly of 4D‐printed microstructures. Adv. Mater..

[26] Hu Y (2015). Laser printing hierarchical structures with the aid of controlled capillary-driven self-assembly. Proc. Natl Acad. Sci. USA.

[27] De Volder M, Hart AJ (2013). Engineering hierarchical nanostructures by elastocapillary self‐assembly. Angew. Chem. Int. Ed..

[28] Duan H, Berggren KK (2010). Directed self-assembly at the 10 nm scale by using capillary force-induced nanocohesion. Nano Lett..

[29] Chandra D, Yang S (2009). Capillary-force-induced clustering of micropillar arrays: is it caused by isolated capillary bridges or by the lateral capillary meniscus interaction force?. Langmuir.

[30] Liu J-L, Feng X-Q (2012). On elastocapillarity: a review. Acta Mech. Sin..

[31] Chandra D, Yang S (2010). Stability of high-aspect-ratio micropillar arrays against adhesive and capillary forces. Acc. Chem. Res..

[32] Chang SW, Chuang VP, Boles ST, Ross CA, Thompson CV (2009). Densely packed arrays of ultra‐high‐aspect‐ratio silicon nanowires fabricated using block‐copolymer lithography and metal‐assisted etching. Adv. Funct. Mater..

[33] Mastrangeli M, Ruythooren W, Celis J-P, Van Hoof C (2010). Challenges for capillary self-assembly of microsystems. IEEE Trans. Compon. Packag. Manuf. Technol..

[34] Li S (2021). Liquid-induced topological transformations of cellular microstructures. Nature.

[35] Benedek M, Wilfling B, Lukas‐Wolfbauer R, Katzur BH, Kaernbach C (2010). Objective and continuous measurement of piloerection. Psychophysiology.

[36] Chaplin G, Jablonski NG, Sussman RW, Kelley EA (2013). The role of piloerection in primate thermoregulation. Folia Primatol..

[37] Küpfer J, Finkelmann H (1991). Nematic liquid single crystal elastomers. Die Makromol. Chem. Rapid Commun..

[38] Yang L, Setyowati K, Li A, Gong S, Chen J (2008). Reversible infrared actuation of carbon nanotube–liquid crystalline elastomer nanocomposites. Adv. Mater..

[39] Yu L (2018). Programmable 3D shape changes in liquid crystal polymer networks of uniaxial orientation. Adv. Funct. Mater..

[40] Barnes M, Verduzco R (2019). Direct shape programming of liquid crystal elastomers. Soft Matter.

[41] Shahsavan H (2020). Bioinspired underwater locomotion of light-driven liquid crystal gels. Proc. Natl Acad. Sci. USA.

[42] Urayama K (2007). Selected issues in liquid crystal elastomers and gels. Macromolecules.

[43] Yusuf Y (2005). Low-voltage-driven electromechanical effects of swollen liquid-crystal elastomers. Phys. Rev. E.

[44] Nobukawa S, Urakawa O, Shikata T, Inoue T (2011). Cooperative dynamics in polystyrene and low-mass molecule mixtures. Macromolecules.

[45] Cai S, Sun Y-C, Ren J, Naguib HE (2017). Toward the low actuation temperature of flexible shape memory polymer composites with room temperature deformability via induced plasticizing effect. J. Mater. Chem. B.

[46] Zhang H (2010). Interpenetrating polymer networks based on acrylic elastomers and plasticizers with improved actuation temperature range. Polym. Int..

[47] Cang Y (2022). On the origin of elasticity and heat conduction anisotropy of liquid crystal elastomers at gigahertz frequencies. Nat. Commun..

[48] Cai W (2023). Optically anisotropic, electrically tunable microlens arrays formed via single-step photopolymerization-induced phase separation in polymer/liquid-crystal composite materials. Light Adv. Manuf..

[49] Vallittu P, Ruyter I (1997). The swelling phenomenon of acrylic resin polymer teeth at the interface with denture base polymers. J. Prosthet. Dent..

[50] Vallittu PK (2009). Interpenetrating polymer networks (IPNs) in dental polymers and composites. J. Adhes. Sci. Technol..

[51] Nishiguchi A, Mourran A, Zhang H, Möller M (2018). In‐gel direct laser writing for 3D‐designed hydrogel composites that undergo complex self‐shaping. Adv. Sci..

[52] Wu Y, Dong X, Kim J-K, Wang C, Sitti M (2022). Wireless soft millirobots for climbing three-dimensional surfaces in confined spaces. Sci. Adv..

[53] Spierings G, Haisma J, Michelsen T (1995). Surface-related phenomena in the direct bonding of silicon and fused-silica wafer pairs. Philips J. Res..

