# Supplementary material for: Artificial-goosebump-driven microactuation
Source: Nat Mater. 2024 Feb 9;23(4):560–9. doi: 10.1038/s41563-024-01810-6 (PMC10990938; doi:10.1038/s41563-024-01810-6)
Supplement: Supplementary file 1 — Supplementary Notes 1–3 and Figs. 1–10. [file 41563_2024_1810_MOESM1_ESM.pdf]

nature materials

Article https://doi.org/10.1038/s41563-024-01810-6

Artificial-goosebump-driven microactuation

In the format provided by the authors and unedited

This PDF file includes:

Supplementary Notes 1 to 3
Supplementary Figs. 1 to 10
Supplementary Video captions 1 to 11

Other Supplementary Materials for this manuscript include the following:

Supplementary Videos 1 to 11

Supplementary Note 1. Mechanical properties of the LCE films

The uniaxial molecular alignment in LCE films imparts them with highly anisotropic mechanical properties (Supplementary Fig. 1a). Under tensile strain, the LCE film exhibits elastic behaviour along its director axis, while its transverse direction displays typical characteristics of plastic materials (Supplementary Fig. 1b). Beyond the elastic range, when the strain surpasses a yield threshold, the LCE film undergoes plastic deformation due to the reorientation of polymer chains, followed by a strain-hardening process until structural failure occurs (Supplementary Fig. 1b).

The introduction of 5CB renders the LCE films more susceptible to ductility. Unlike the elastic response of the LCE without 5CB dopant along the director in a wide strain range, a decreased yield point is observed for 5CB-doeped LCE film, indicated by the remarkably increased dissipation factor (tanδ) calculated from the loading-unloading hysteresis loops at higher strains (Supplementary Fig. 1c). As depicted in Supplementary Fig. 1d, a moderate addition of 5CB (below 50%) appears to have no significant impact on the Young's modulus (E) within the elastic region (E = 7.4 MPa for the undoped LCE; E = 7.5 and 8.6 MPa for LCEs with 25% and 50% doping, respectively), while their yield points and yield strengths decrease with increasing 5CB content. In contrast, excessive doping (e.g., 75%, resulting in E = 2.1 MPa) leads to a substantially lower yield point and yield stress, resulting in increased ductility and leading to plastic deformation. This makes the LCE films challenging to be handled for subsequent applications. Moreover, this easy ductility could also lead to a poor long-term actuation stability of microstructures as considerable stress may be generated in the laser-induced goosebumps. Consequently, it is not recommended to exceed a 50% doping content with 5CB, and we have opted for a 25% dopant for our subsequent application unless otherwise stated.

Supplementary Note 2. Microstructure actuation enabled by the fs laser

The actuation of the microstructures was also conducted inside the Nanoscribe system by programming the integrated femtosecond (fs) laser (Nanoscribe GmbH, centre wavelength: 780 nm, pulse duration: 80 fs, repetition rate: 80 MHz). Femtosecond lasers are known for their exceptional heating speed, owing to their ultrashort bursts of high-intensity light. To investigate the response time of our microactuation system, we adjusted the camera's exposure time to 10 ms and captured the motion of a

microhair actuated by the fs laser. By relocating the laser spot alternately between the two sides of the microactuator, we observed that the microhair deflected in the subsequent frame (10 ms later), which indicates the utilization of the fs laser empowers our microactuation system to achieve a swift response time within 10 ms.

Achieving perfectly synchronized motions among different microstructures using a single laser source can be challenging at the physical level. However, we can attain a state of *quasi-synchronous* actuation of the fs laser for these microactuators at the microscale, by considering the high scanning speed, reaching up to 10^5 µm/s. For example, when we generate an array of laser spots with diameters of 1 µm while scanning at a moderate speed of 10^4 µm/s, the time interval between individual spots is approximately 0.1 ms. This time difference is significantly faster (approximately two orders of magnitude) than the response time of the microstructures' actuation. We recorded the laser using a frame rate of 10 Hz, which confirmed that these laser spot arrays were positioned on the LCE skin within the 10 ms frame duration (see Supplementary Fig. 5). Consequently, these laser spots can be considered as effectively applied simultaneously to the LCE skin.

Besides, the total processing time required to open each assembled microstructure in Fig. 6 currently is approximately 0.88 seconds (Supplementary Video 11). This duration encompasses three essential steps: the time dedicated to actuating the assembly, stage movement to reach the next targeted assembly, and the automated interface finding process. Among these steps, the most time-consuming elements are the stages of moving the stage and conducting subsequent interface finding. Notably, the actual disassembly of the microstructure itself constitutes only a fraction of the overall timeframe. To expedite processing and enhance information writing efficiency, we can optimize the laser programming codes by reducing the frequency of stage movements and automated interface finding and incorporating Galvo scanning mode for the laser. The Galvo scanner integrated into the laser system plays a pivotal role in swiftly steering the laser beam across the substrate's xy-plane, thereby facilitating high-speed writing within each addressable view field. During each stage movement, followed by a single automated interface finding step, the laser traverses and rapidly actuate these assemblies within the view field at an high speed (optimized key code using Galvo scanners: “X(/Y)offset *n*”, where '*n*' represents the distance between each pixel). Consequently, a substantial number of pixels within the field of view can be

disassembled concurrently. This strategic optimization eliminates the need for repetitive stage movements and automated interface finding for each individual pixel. As a result of these measures, the processing time for each pixel has been notably reduced to approximately 0.20 seconds, significantly enhancing the efficiency of our microstructure disassembly process.

Supplementary Note 3. Controllable micro-mirrors for light steering

Our microactuation systems achieve precise manipulation of the reflective plane for light steering, catering to both subtle and large steering angles. Micro-mirrors with precise control over small steering angles are essential for high-resolution applications, such as laser-based spectroscopy and microscopy, where precise control of the reflective plane is necessary to achieve accurate measurements and observations. As shown in Extended Data Fig. 4a, the degree of tilting angle (α) of the reflection plane increases as we enhance the scanning powers or diameters of the laser spots, which amplifies the laser dosage or heating areas required for generating enlarged artificial goosebumps and higher uplifts. Our FE simulation (Fig. 5d) corroborates these results, aligning with the experimental results (Supplementary Video 6). In addition, we provide a solution for achieving large steering angles of the reflective plane by uniformly scaling down the micro-mirrors (Extended Data Figs. 4b and 4c). The micro-mirrors are miniaturized, while the generated artificial goosebump remains the same, which induces more pronounced deformations to the micro-mirrors and consequently augmenting the plane's tilt. Additionally, we can also achieve free tilting direction (0-360°) of the mirror plane through the *quasi-synchronous* actuation of laser spots (Supplementary Note 2, Supplementary Fig. 5). A single laser spot can cause the individual supporting pillars of the micro-mirror to lift up and tilt the mirror plane toward the direction of the activated pillar (Fig. 5d), while simultaneous actuation of two spots on adjacent pillars can enable the tilt direction between these two pillars (Extended Data Fig. 4d). By rationally tuning the dosage (thus obtaining different geometries of artificial goosebumps) of the two laser spots acting on any two adjacent pillars, we can realize free rotation of the mirror plane with controllable angular tilt.

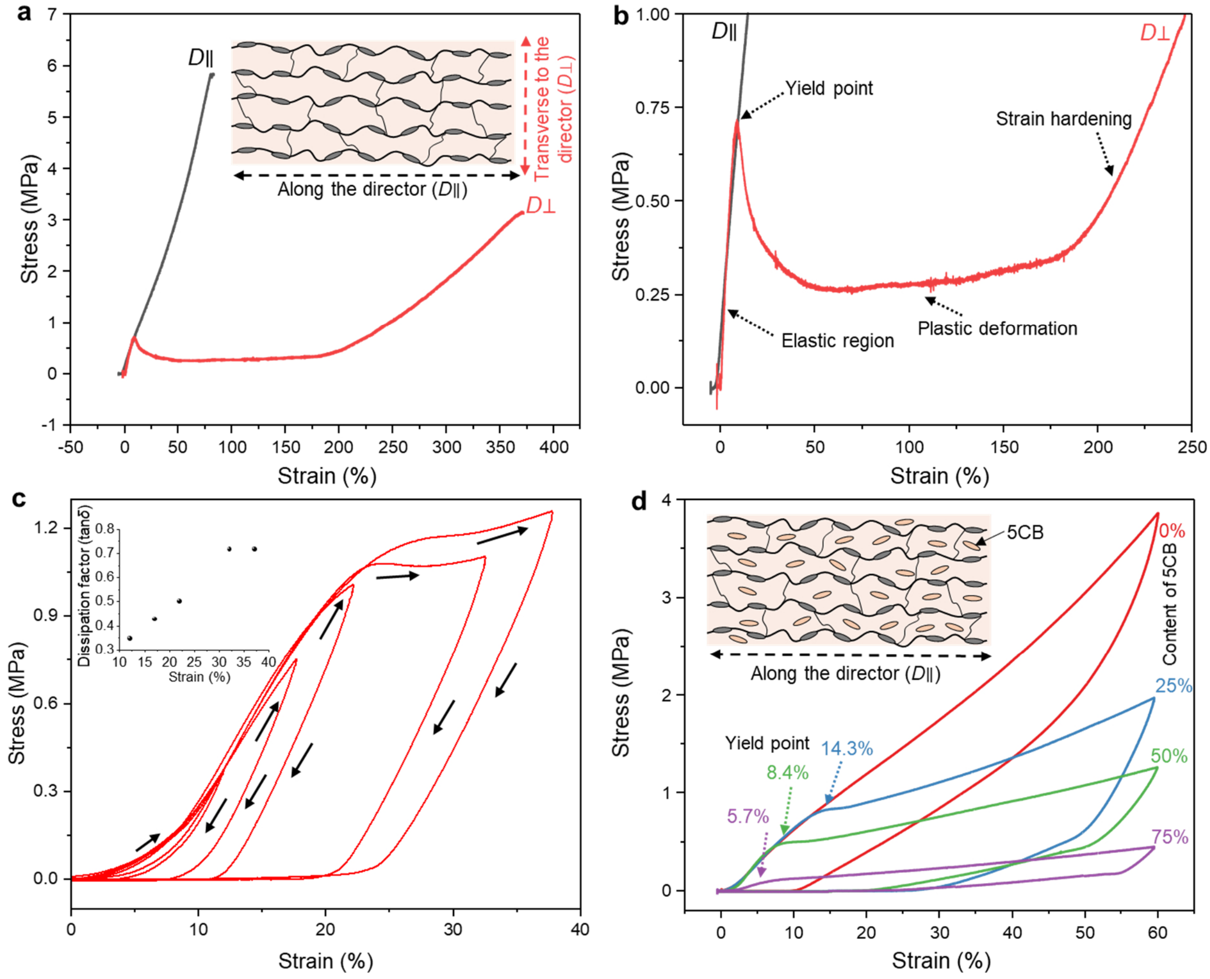

Supplementary Fig. 1 Mechanical properties of the LCE materials. a, Strain-stress curves of LCE films (without doping) measured along (D∥) and transverse to (D⊥) the director direction. **b**, Detailed strain-stress curves showing the anisotropic mechanical properties of the LCE films (without doping). **c**, Hysteresis loops of LCE film with a 25% dopant of 5CB at various strains. Inset shows the corresponding dissipation factors (tan*δ*) across different strains. **d**, Strain-stress curves of LCE films with varying content of 5CB doping under a 60% strain of loading-unloading test.

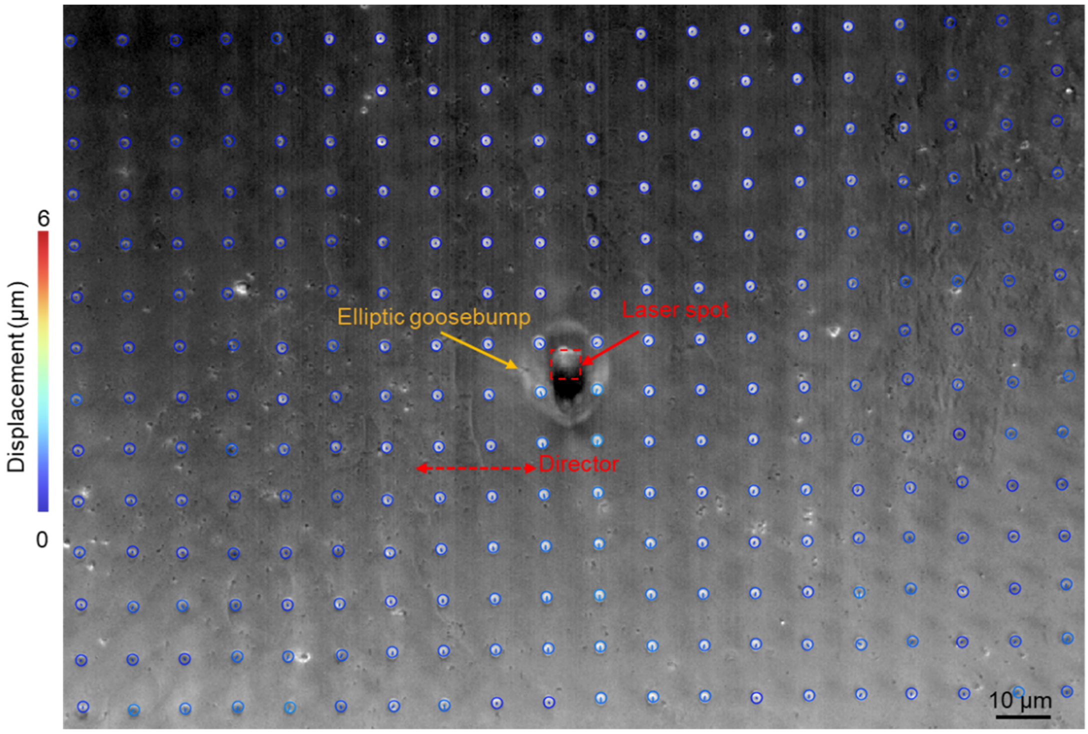

Supplementary Fig. 2 Experimental tracking of surface displacement on an LCE surface with a 25 wt% 5CB dopant actuated by a laser with a spot canning area of 5×5 μm². When mounting the director of LCE in the *x* direction of the camera view, the short-axis of the generated artificial goosebump aligns along the director (in the *x* direction of the camera view).

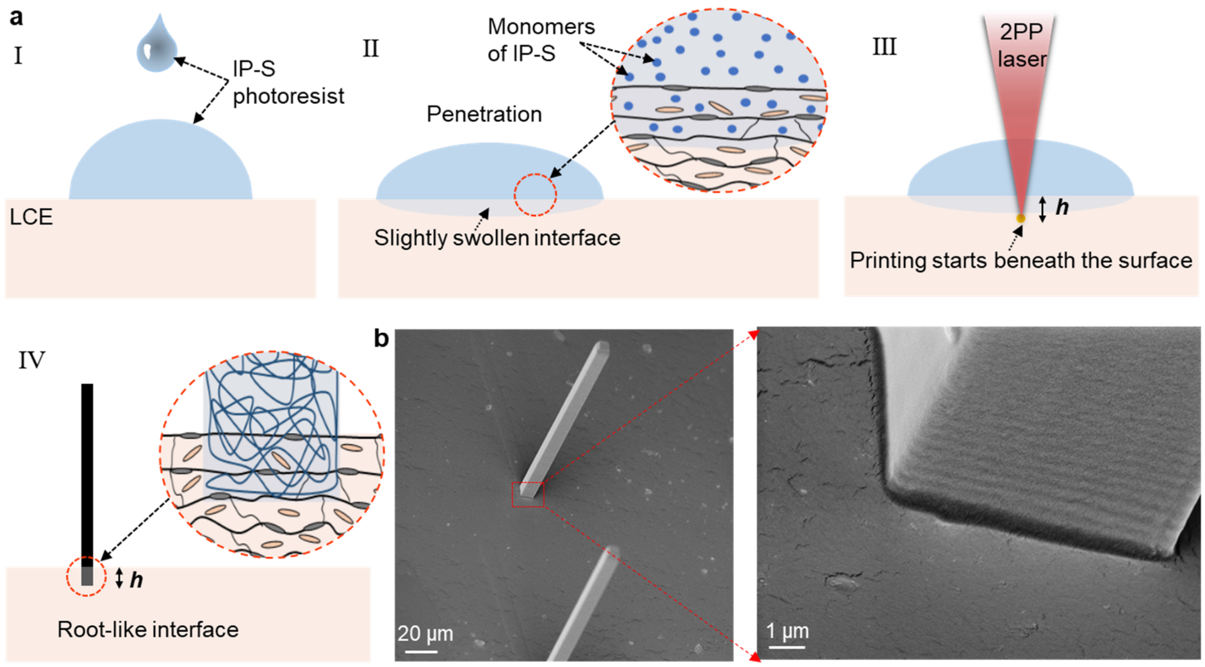

Supplementary Fig. 3 Strong interfacial bonding between the 3D printed microstructures and the LCE surface. a, Schematic illustration showing the key steps for ensuing a robust interfacial bonding between microstructures and the LCE surface. **b**, SEM images of a micropillar printed on the LCE surface and their interface.

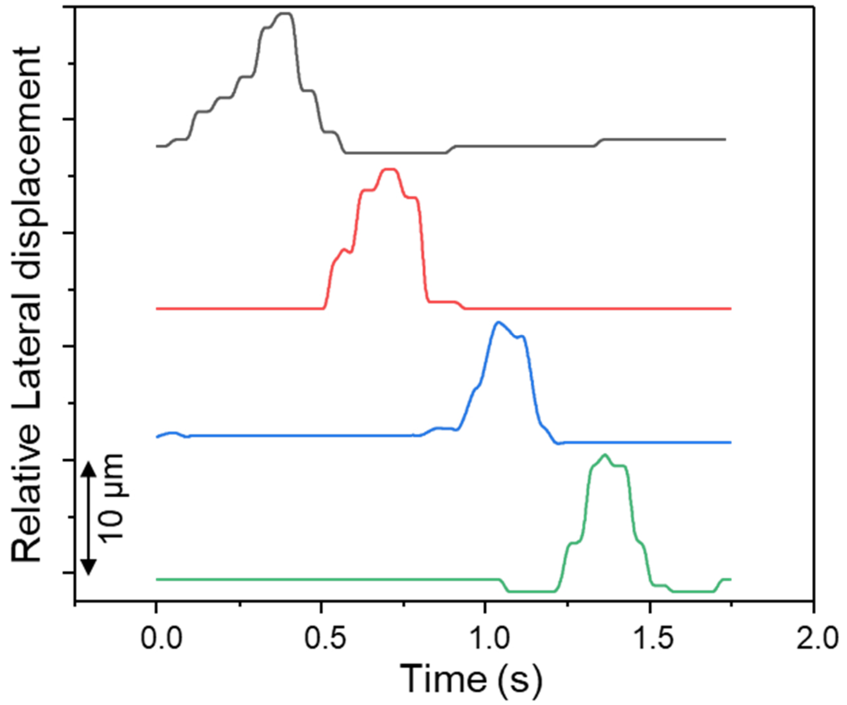

Supplementary Fig. 4 Lateral tip displacements (along the horizontal direction of the view) of four microhairs in a row as the laser linearly sweeps besides them (refer to Fig. 4h).

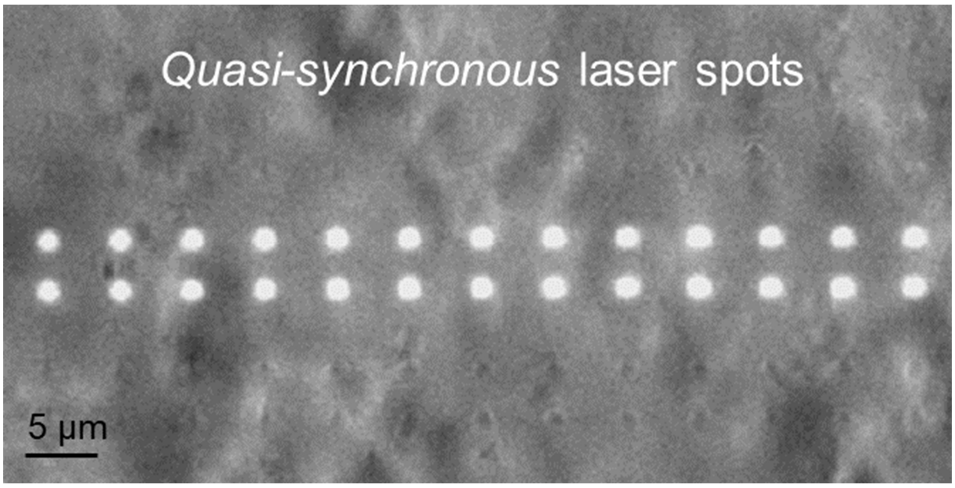

Supplementary Fig. 5 Optical image showing the fast scanning speed (10^4 μm/s) of a single laser source could allow the *quasi-simultaneous* exposure of laser spots on the LCE skin.

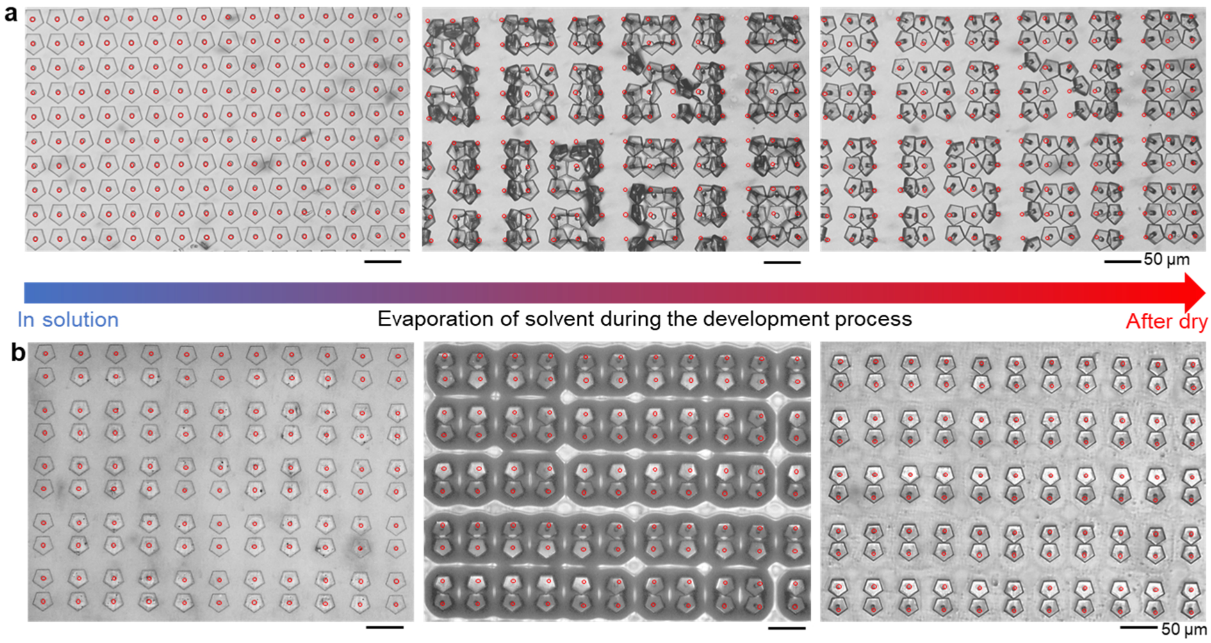

Supplementary Fig. 6 Comparing the self-assemblies of mushroom-like mirrors with uniform (a) and non-uniform (b) spacing during the development process. A uniform spacing generates a random assembly because of the equal attaching possibility of the mirror to their surroundings (**a**), while introducing an additional spacing (*Δd*) between two paired mushroom structures enables a uniform bi-assemblies across large areas (**b**). The red dots mark the pillar bottom position of the mirrors to indicate the displacement of the top mirror and bottom pillar induced by capillary force.

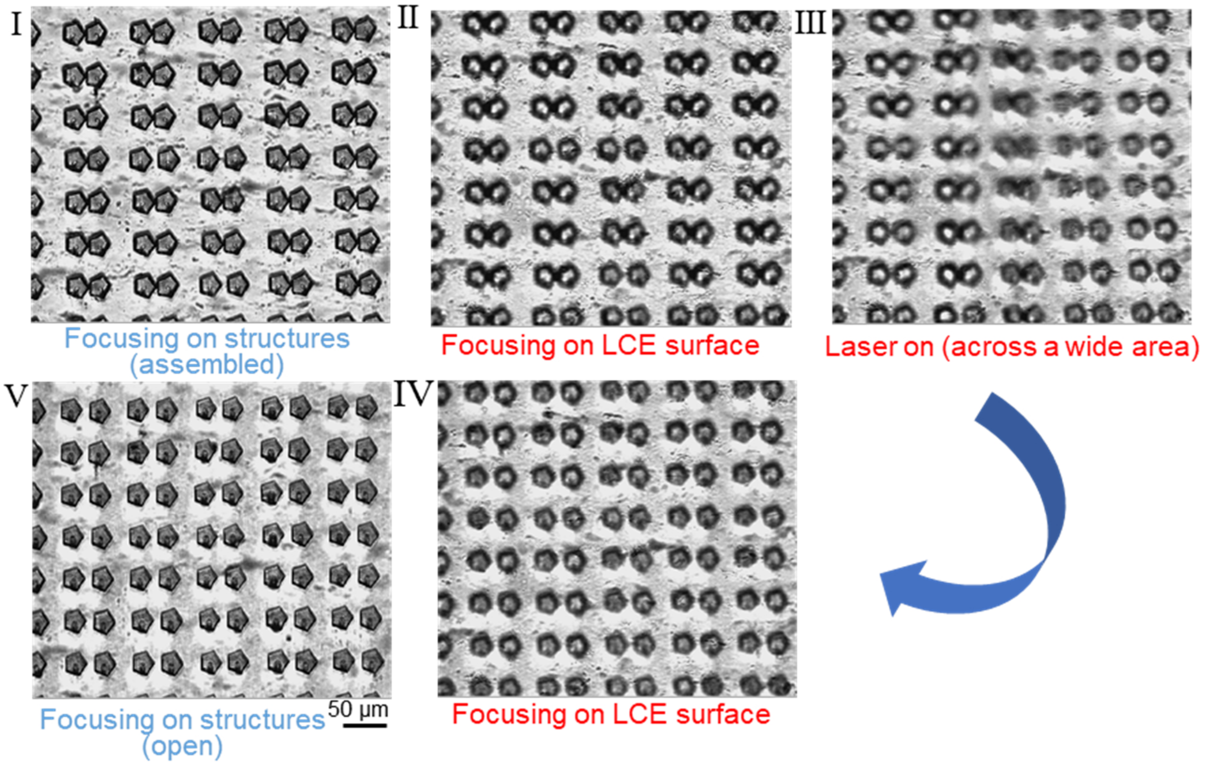

Supplementary Fig. 7 Optical images showing the globally disassembling process of uniformly paired bi-assemblies inside the Nanoscribe system (recorded by AxioVision Live-View camera).

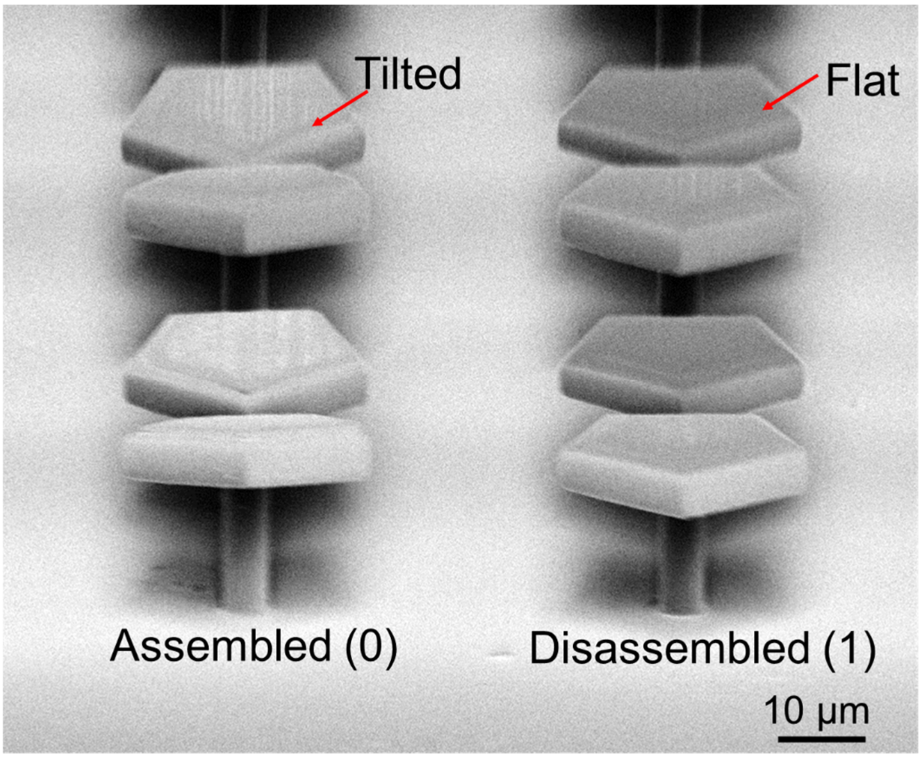

Supplementary Fig. 8 Scanning electron microscopy (SEM) image showing the morphologies of paired mirrors before (0) and after (1) disassembly.

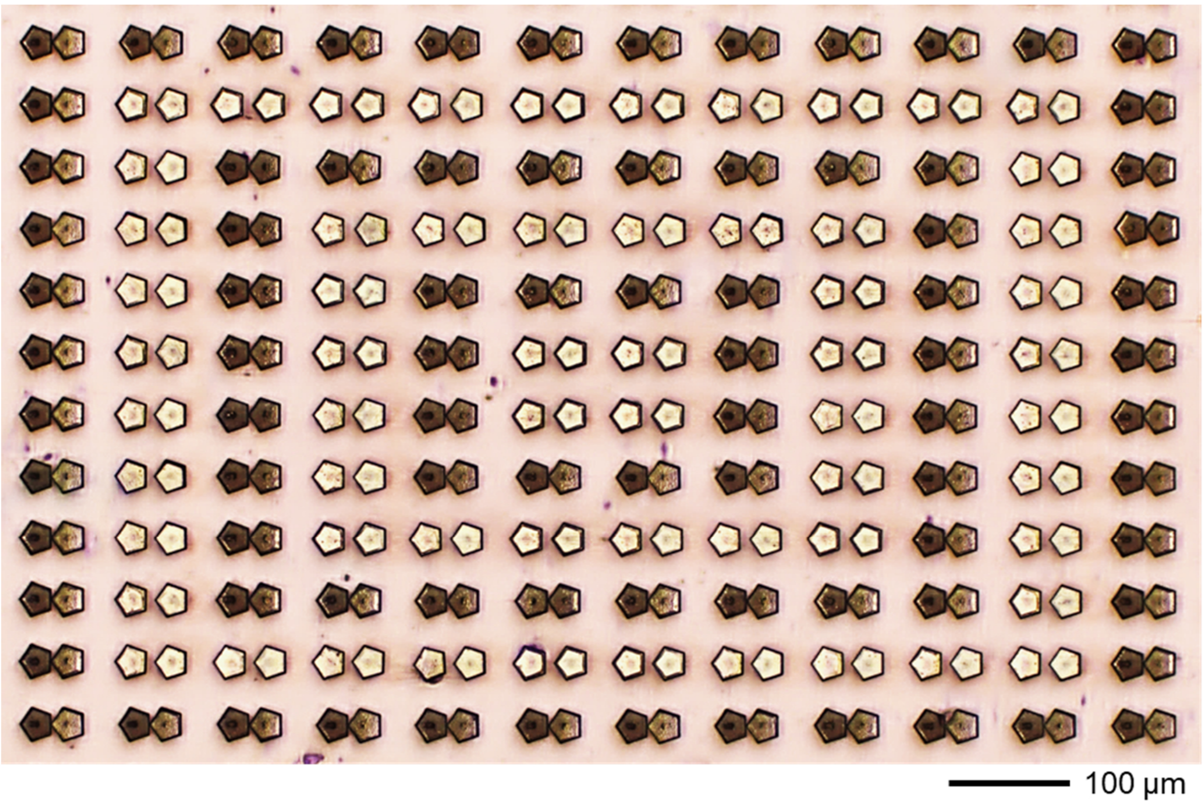

Supplementary Fig. 9 Optical image showing a written pattern composed of alternating assembled (dark) and disassembled (bright) rectangles.

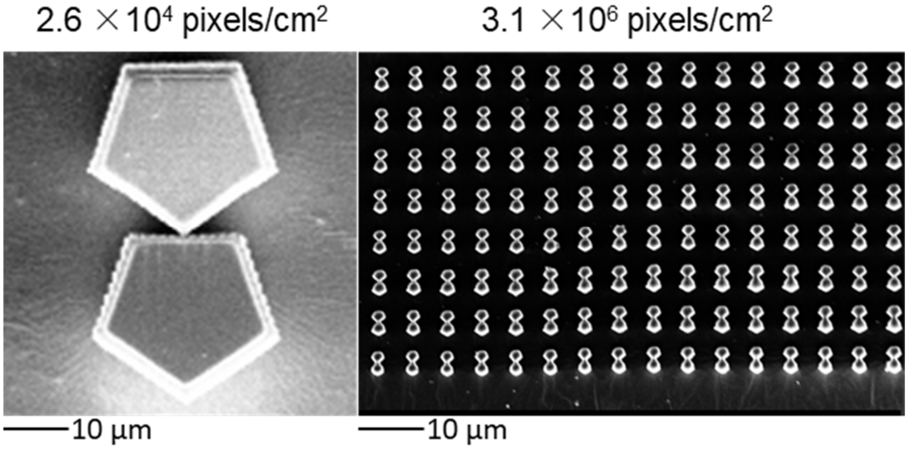

Supplementary Fig. 10 SEM images that showcase the scaled-down pixels (right) for comparison with the current pixels we demonstrate in Fig. 6 (left).

Supplementary Video captions 1 to 11

Supplementary Video 1: Muscle-like LCE film showing fast deformation of contraction and expansion due to air-blowing induced temperature fluctuation (heated at 100 ^{o}C, in real-time).

Supplementary Video 2: Comparison of laser-induced in-plane deformation of the LCE with 5CB dopant of 0 and 50 wt% (in real-time).

Supplementary Video 3: Light-fueled passive microhairs on the LCE (in real-time).

Supplementary Video 4: Sequentially actuation of microhairs powered by linearly sweeping of laser (in real-time).

Supplementary Video 5: 0-360° rotation of microhairs powered by circular sweeping of laser (in real-time).

Supplementary Video 6: Artificial goosebump-driven tilting of a micro-mirror on the LCE skin (in real-time).

Supplementary Video 7: Self-assembly of printed large-aspect-ratio micropillars due to evaporation-induced capillary force (in real-time).

Supplementary Video 8: Laser-enabled globally disassembling process of self-assembled microstructures including bi-assemblies, tetra-assemblies, and other assemblies (in real-time).

Supplementary Video 9: Comparison of self-assembled mushroom-like mirror arrays with uniform spacing design (d) and nonuniform spacing design (in real-time).

Supplementary Video 10: Laser-enabled global disassembly process of uniform bi-assembly arrays (5 times faster).

Supplementary Video 11: Programmable disassembling process for selectively opening bi-assembly pixels for information storage (in real-time).
